# Time-series prediction of adverse birth outcomes in the U.S. using multilayer perceptron neural networks

**DOI:** 10.1371/journal.pdig.0001515

**Published:** 2026-07-01

**Authors:** Bayuh Asmamaw Hailu

**Affiliations:** Monitoring and evaluation, Wollo University, Dessie, Ethiopia; China Medical University, CHINA

## Abstract

Adverse birth outcomes (ABOs), including preterm birth, low birth weight, low Apgar scores, and neonatal mortality, remain major public health challenges in the United States and disproportionately affect racial, socioeconomic, and demographic subgroups. A time-series multilayer perceptron (MLP) neural network was developed to predict ABOs and identify populations with elevated risk using U.S. Vital Statistics Natality Birth Data from 2009 to 2023, covering 57.9 million births. Unlike standard methods such as ARIMA, the MLP model captures nonlinear relationships among predictors and learns seasonal patterns directly from the data. Key predictors included maternal age, body mass index, adequacy of prenatal care, education, race/ethnicity, and short interpregnancy intervals. Monthly aggregated ABO rates were modeled using data from January 2009 to December 2022 (168 monthly observations), while data from 2023 (12 monthly observations) were reserved as an independent holdout test set. Predictive performance on the holdout set was assessed using root mean square error (RMSE = 0.181), and forecasts were projected through 2030. Between 2009 and 2023, ABO prevalence increased from 14.9% to 15.5%, with Black and American Indian/Alaska Native mothers consistently exhibiting the highest rates. Pregnancy-related medical conditions, advanced maternal age, underweight status, low educational attainment, and short birth intervals were among the variables most strongly associated with model predictions. Projections suggest continued increases in ABO risk among underweight mothers and American Indian/Alaska Native populations. Performance was lower for smaller or structurally disadvantaged subgroups, highlighting challenges in predicting outcomes for high-risk populations. These findings demonstrate that time-series MLP models can support forecasting of adverse birth outcomes and help identify groups experiencing elevated risk. Predictive models may support public health surveillance, maternal health planning, and resource allocation, although forecasts should be interpreted cautiously and are not intended for individual-level clinical decision-making.

## Author summary

This study looked at why some babies in the U.S. are born too early, too small, or with health problems. The researcher used birth records from nearly 58 million babies born between 2009 and 2023. A multilayer perceptron (MLP) neural network model was developed to predict future trends.

The model shows that the overall rate of these problems will go up slowly through 2030. The highest risks are for underweight mothers and for American Indian/Alaska Native mothers. The model works well for large groups but is less reliable for small, high risk groups.

These findings can help public health officials plan better prenatal care, nutrition programs, and family planning services. This also shows that caution is needed when using predictions for very small or disadvantaged groups.

## Introduction

Adverse birth outcomes (ABOs), including preterm birth, low birth weight, low Apgar scores, and neonatal mortality, are significant public health challenges worldwide and in the United States [1,2]. These outcomes affect the long-term health and development of newborns and contribute to health disparities among racial, socioeconomic, and demographic groups [2,3]. Globally, approximately 15 million infants are born preterm each year, and the burden is disproportionately higher among marginalized populations [1,2].

In the U.S., Black and American Indian/Alaska Native (AIAN) mothers experience consistently higher rates of ABOs compared with other racial groups, reflecting structural inequities in healthcare access, prenatal care, and social associated characteristics of health [4,5]. Advanced maternal age, inadequate prenatal care, smoking, short interpregnancy intervals, and maternal underweight status are well-established predictive variables for ABOs [3,4].

Traditional epidemiological approaches have improved understanding of ABO predictive variables, yet they may fail to capture complex, nonlinear interactions among variables. Machine learning (ML), particularly time series models using multilayer perceptron (MLP) neural networks, can analyze high-dimensional data and identify previously unrecognized patterns [6]. Previous studies have demonstrated the feasibility of artificial intelligence approaches for predicting clinical requirements and outcomes using Intensive Care Unit (ICU) databases [7]. This gives policymakers and providers a more flexible way to forecast trends and spot high-risk populations, especially across different subgroups where standard methods have trouble [8].

This study leverages U.S. Vital Statistics Natality Birth Data (2009–2023) to develop a time-series MLP model for three related but distinct aims: (1) to forecast national trends in ABOs through 2030, (2) to identify high-risk subgroups (by race, body mass index (BMI), education, prenatal care, and marital status), and (3) to measure the predictive contribution of individual variables associated with adverse birth outcomes, including maternal age, pregnancy-related medical conditions, and birth interval predictive variables [1,2,6].

## Methods

### Study design and data source

A population-based study using repeated cross-sectional natality data was conducted using U.S. Vital Statistics Natality Birth Data from 2009 to 2023. The dataset included maternal demographic characteristics, health factors, prenatal care metrics, and birth outcomes for 57.9 million births (average 3.86 million annually) (see S1 Text) [9].

### Data preprocessing and harmonization

The raw natality files for 2009–2023 were obtained from the NCHS. Missing values for any predictor or outcome variable were excluded listwise, affecting less than 2% of births per year. All variables were recoded into binary or categorical form. Variable definitions were checked for consistency across the 15 years. The following changes were noted: (1) Prenatal care adequacy was defined through the number of prenatal visits (<5 vs. >5), which remained consistent after 2003. (2) BMI was calculated from prepregnancy weight and height using the same formula each year; underweight was defined as BMI < 18.5 kg/m^2^. (3) Cigarette smoking was recorded as any smoking during pregnancy (yes/no) and did not change coding. (4) Pregnancy-related medical risk were a composite indicator and were consistently available from 2009 onward. No birth certificate revisions between 2009 and 2023 altered these specific items according to NCHS documentation. For any variable with minor definitional shifts (e.g., race categories), the harmonization steps are detailed in S1 Text.

### Outcome variables

**Fig 1:**
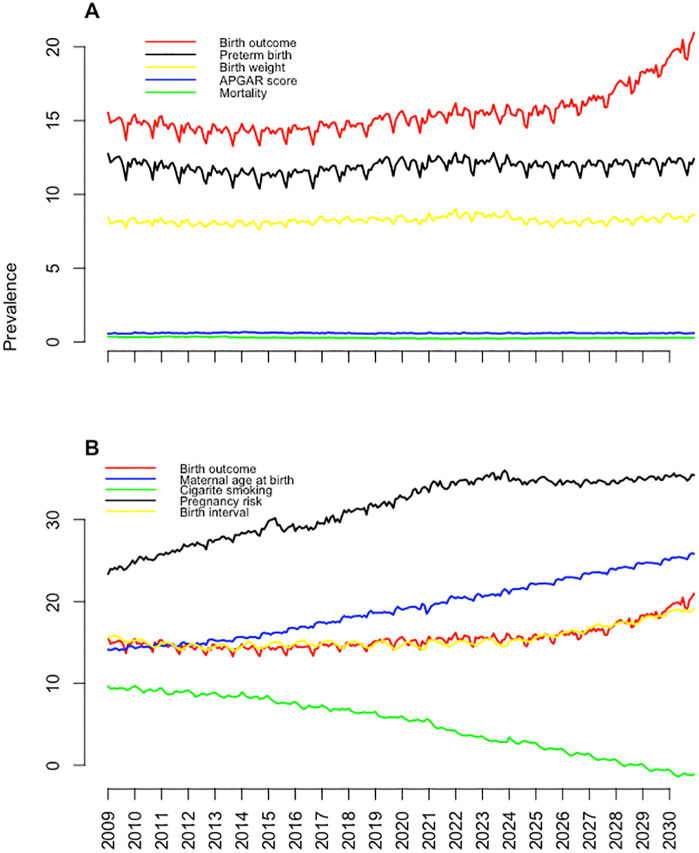
Time-series analysis of overall ABOs using an MLP model. **(A)** ABO components (preterm birth, low birth weight, low Apgar score, neonatal mortality). **(B)** Influencing factors (maternal age, BMI, prenatal care, pregnancy-related risks).

**Fig 2:**
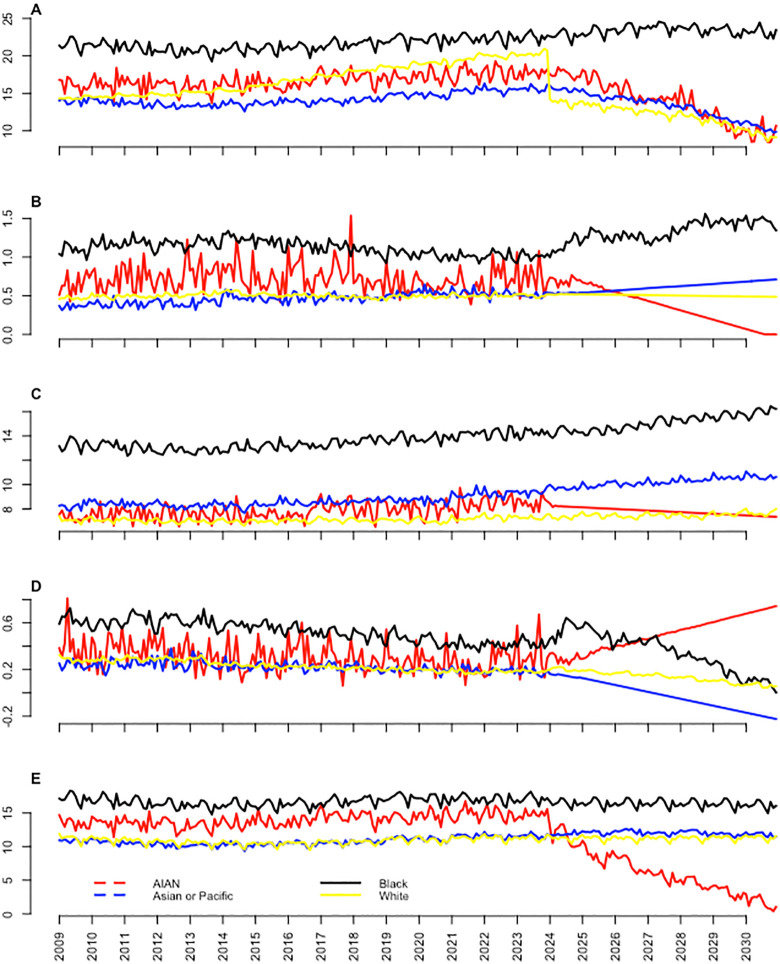
Trends and forecasts of ABOs by race: (A) Overall ABOs, (B) 5-minute Apgar score, (C) Birth weight, (D) Mortality, (E) Preterm birth.

**Fig 3:**
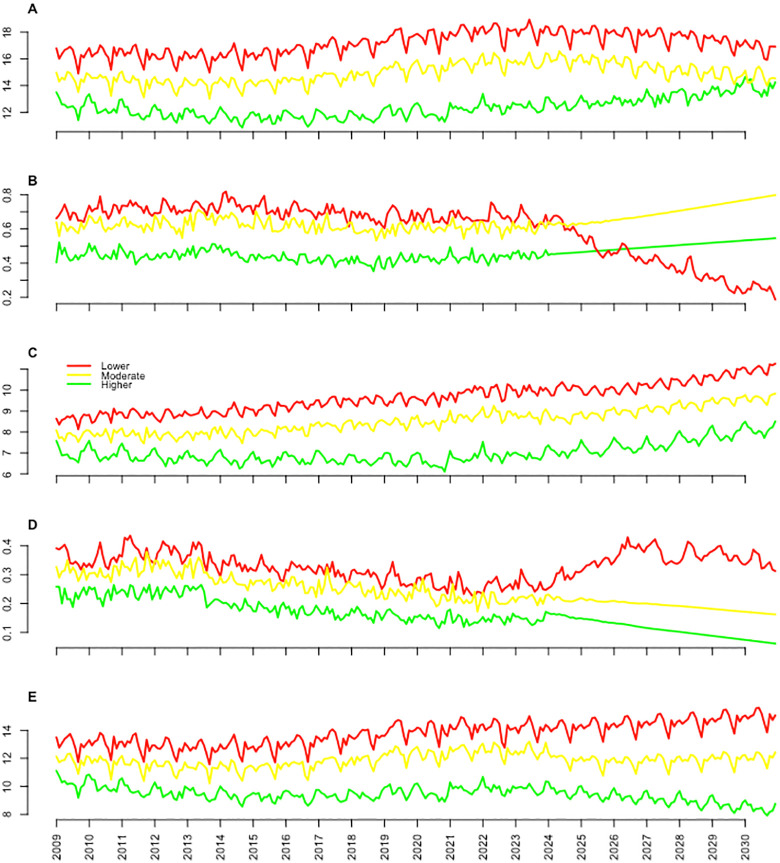
Trends and predictions of ABOs by maternal education level: (A) Overall ABOs, (B) low 5-minute Apgar score, (C) Low birth weight, (D) Mortality, (E) Preterm birth.

**Fig 4:**
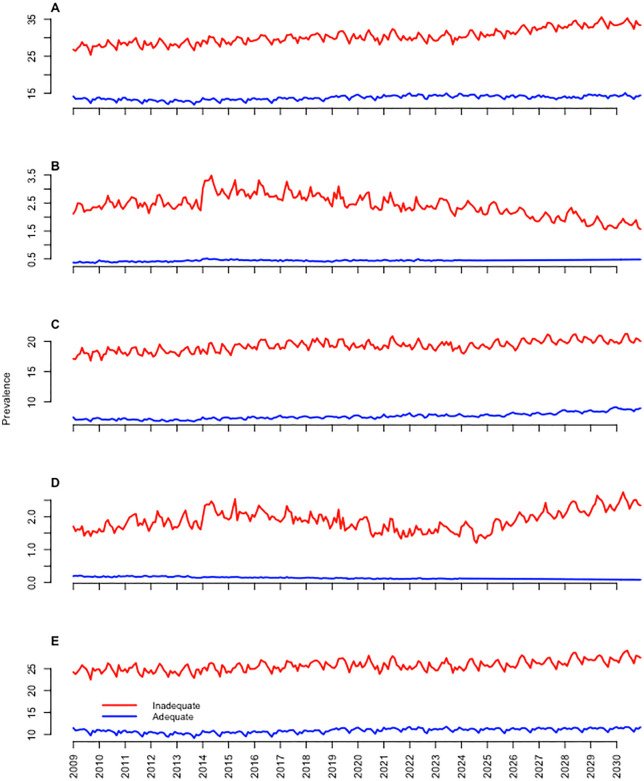
Trends and predictions of ABOs by prenatal care adequacy: (A) Overall ABOs, (B) Low 5-minute Apgar score, (C) Low birth weight, (D) Mortality, (E) Preterm birth.

**Fig 5:**
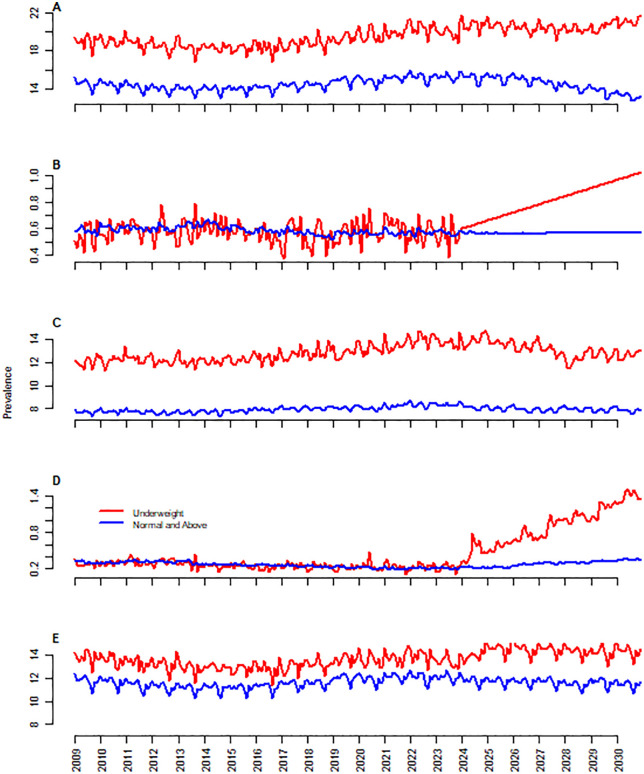
Trends and predictions of ABOs by maternal BMI: (A) Overall ABOs, (B) Low 5-minute Apgar score, (C) Low birth weight, (D) Mortality, (E) Preterm birth.

The primary outcome was a composite adverse birth outcome (ABO), defined as the occurrence of one or more of the following conditions: preterm birth (<37 weeks’ gestation), low birth weight (<2.5 kg), a low 5-minute Apgar score (0–3), or neonatal mortality. The composite was chosen for population-level surveillance and forecasting, not for clinical severity ranking. The four components often co-occur, for example, a preterm infant is more likely to have low birth weight. Overlapping cases were counted only once in the composite outcome. Each component was also modeled separately (Figs 1–5, and S7–S12 and S1 Text) to avoid masking component-specific trends [1,10].

### Predictor variables

Predictors included maternal age, BMI, prenatal care adequacy, race/ethnicity, education, cigarette smoking, pregnancy-related medical risks, and birth interval (<2 years). Contribution of each predictor to model performance was assessed using the Drop-One-At-A-Time method with RMSE as the evaluation metric (S1 Text and S1 Table) [11,12]. This method measures predictive contribution (how much RMSE changes when a predictor is removed), not causal effect.

### Machine learning model

The main reason to use MLP instead of standard time-series models (like ARIMA or exponential smoothing) is that MLP automatically picks up nonlinear interactions between predictors without requiring the analyst to guess them ahead of time. This is useful here because ABO predictive variables (age, BMI, race, prenatal care) interact in complicated ways. A time-series multilayer perceptron (MLP) neural network was used to predict adverse birth outcomes (ABOs) and to forecast trends through 2030 [13]. The model included 28 input nodes representing four primary independent variable domains (architecture shown in S3 Fig), five hidden layers to capture nonlinear relationships, and a single output layer for ABO prediction. Each hidden layer contained 12 nodes (determined by the lag-12 ACF). The activation function for all hidden layers was the hyperbolic tangent (tanh); the output layer used a linear activation function for continuous prediction. Optimization was performed using the Adam algorithm with a learning rate of 0.001. No regularization was applied. Training stopped after 50 repetitions (epochs) with no early stopping, as the RMSE on the validation set stabilized by 30 epochs. Hyperparameters (number of hidden layers, nodes per layer, learning rate, and repetitions) were selected by testing a grid: hidden layers [3, 5, 7], nodes [6, 12, 24], learning rates [0.0005, 0.001, 0.005], and repetitions [30, 50, 100]. Hyperparameter tuning and architecture selection were conducted exclusively using the 2009–2022 training period. The 2023 holdout dataset was not accessed during model development and was used only once for final out-of-sample evaluation. The final configuration was selected using comparative predictive performance within the 2009–2022 training period only, while the 2023 holdout dataset remained untouched until final evaluation. All model code and tuning details are available at https://github.com/Bayuh23/MLP. Model training employed back propagation with weight optimization to minimize the root mean squared error (RMSE) [12,14]. RMSE was selected because the outcome represented continuous monthly ABO rates rather than binary individual-level outcomes, and it appropriately penalizes larger forecasting errors. The number of input nodes varied slightly in subgroup analyses depending on predictor availability, with group-specific model architectures illustrated in S4 Fig.

Four modeling approaches were evaluated, including a neural network without hidden layers (AR(1) analogue), a neural network with 12 lag inputs (AR(12) analogue), an automatic time-series model selection approach, and a deep learning–based MLP for time-series forecasting [15]. The deep learning MLP achieved the lowest overall RMSE (0.181) and was therefore selected for the final analyses. The improvement over the AR(1) analogue (RMSE 0.266) and the AR(12) analogue (RMSE 0.274) shows that capturing nonlinearity and deeper time patterns helps for this prediction problem. Model predictions were stratified by race, maternal BMI, prenatal care adequacy, marital status, and educational attainment to assess subgroup disparities (S1 Table).

### Statistical analysis

To address the three study aims separately: (1) For national trend forecasting, the MLP model was trained on monthly aggregated ABO rates from 2009 to 2022 and used to generate point forecasting for 2024–2030. (2) To identify high-risk subgroups, the same model architecture was applied separately to subgroups defined by race, BMI, education, prenatal care adequacy, and marital status, with subgroup-specific forecasts and RMSE reported. (3) To measure predictive contributions of individual predictive variables, the drop-one-variable-at-a-time method was used, comparing RMSE changes when each predictor was removed.

The unit of analysis for the time series models was the month. Individual birth records were aggregated into monthly ABO rates from January 2009 to December 2023, resulting in 180 monthly observations. Seasonality and lag structures were evaluated using the autocorrelation function (ACF) analysis and time-series decomposition (S1 and S2 Figs) [16]. The dataset was split into a training period covering 2009–2022 and an independent holdout testing period consisting of data from 2023. No information from the 2023 holdout period was used for final model evaluation until model development and configuration selection were completed. Forecasts for adverse birth outcomes were generated for the period 2024–2030 using the selected MLP model. Data preprocessing, model training, and prediction were performed in R using the “*nnfor”* package, while data management and descriptive analyses were conducted in STATA [17].

## Results

### Seasonality and descriptive trends of adverse birth outcomes

Analysis of U.S. Vital Statistics data from 2009–2023 revealed that adverse birth outcomes (ABOs) exhibited seasonal patterns, with peaks at the start and end of each year, suggesting higher risk during winter months, and a secondary peak in summer, consistent with higher preterm birth rates (S1 Fig and S2 Text). The seasonal peaks were about 0.3 to 0.5 percentage points above the yearly average, with the highest rates in January and December.

### Population characteristics and ABO trends

Between 2009 and 2023, 57.9 million births occurred, with ABO prevalence increasing from 14.9% to 15.49%. Mortality rates decreased from 33 per 10,000 births in 2009–22 per 10,000 births in 2023. ABOs were highest among Black and AIAN mothers, mothers with low BMI, lower education, and inadequate prenatal care (Table 1).

**Table 1:** Descriptive statistics of registered births, adverse birth outcomes (ABOs), influencing factors, and demographic distributions (2009–2023).

I. Total registered births and percentage of ABOs within high-risk groups						
Year	Total birth	APGAR5 <4(%)	Deaths (%)	Low birth weight (%)	Preterm (%)	Adverse birth outcome (%)
2023	3605081	0.58	0.22	8.58	12.23	15.49
2022	3676029	0.59	0.22	8.6	12.2	15.45
2021	3669928	0.59	0.22	8.52	12.28	15.41
2020	3619826	0.58	0.23	8.24	11.97	15.02
2019	3757582	0.58	0.24	8.31	12.15	15.15
2018	3801534	0.57	0.26	8.28	11.71	14.65
2017	3864754	0.6	0.26	8.27	11.63	14.6
2016	3956112	0.61	0.27	8.16	11.39	14.35
2015	3988733	0.61	0.28	8.07	11.28	14.23
2014	3998175	0.63	0.29	8	11.31	14.25
2013	3940764	0.63	0.33	8.02	11.38	14.22
2012	3960796	0.6	0.34	7.99	11.54	14.3
2011	3961220	0.6	0.34	8.09	11.72	14.52
2010	4007105	0.59	0.32	8.14	11.98	14.81
2009	4137836	0.56	0.33	8.16	12.17	14.9
Total	57945475					
Average	3863031					
II. Registered births by classification (proportional distribution)						
Year	Education (%) Low/ Moderate/ High		Unmarried (%)	Race (%) 1/ 2/ 3/ 4*		PNC < 5 (%)
2023	38.23/ 25.61/ 36.16		40.21	73.92/ 15.4/ 0.97/ 9.71		6.48
2022	37.72/ 26.52/ 35.75		39.98	73.8/ 15.62/ 0.97/ 9.61		6.46
2021	37.27/27.14/35.59		40.11	73.93/ 15.73/ 0.95/ 9.39		6.14
2020	38.38/ 27.64/ 33.98		40.77	73.29/ 16.17/ 0.97/ 9.57		6.03
2019	38.39/ 28.1/ 33.51		40.33	73.27/ 15.99/ 0.97/ 9.76		5.76
2018	38.52/ 28.43/ 33.06		39.98	73.58/ 15.83/ 0.96/ 9.64		5.54
2017	38.83/ 28.79/ 32.38		40.09	72.98/ 16.21/ 0.94/ 9.87		5.54
2016	38.97/ 28.99/ 32.04		39.76	73.56/ 15.79/ 0.95/ 9.7		5.38
2015	39.63/ 29.38/ 31		40.22	74.67/ 15.34/ 0.97/ 9.02		5.27
2014	40.23/ 29.5/ 30.27		40.21	74.82/ 15.25/ 1.01/ 8.92		5.25
2013	40.82/ 29.35/ 29.83		40.55	75.97/ 16.12/ 1.17/ 6.75		9.68
2012	41.88/ 28.93/ 29.2		40.69	75.92/ 16.02/ 1.16/ 6.89		9.49
2011	43.18/ 28.53/ 28.28		40.64	76.43/ 15.98/ 1.17/ 6.41		9.46
2010	45.38/ 27.69/ 26.93		40.82	76.78/ 15.89/ 1.17/ 6.17		9.43
2009	47.7/ 27.03/ 25.27		40.98	76.85/ 15.9/ 1.18/ 6.07		9.73
III. Proportion of high-predictive variables						
Year	Age >34 (%)	Smoker (%)	Pregnancy risk (%)	Birth interval <2y (%)	Low BMI (%)	
2023	20.91	3	35.39	14.99	2.68	
2022	20.49	3.67	34.7	14.95	2.69	
2021	19.87	4.6	34.2	14.91	2.68	
2020	19.21	5.54	33.26	14.61	2.82	
2019	18.75	5.94	32.04	14.17	3.04	
2018	18.3	6.48	31.32	14.74	3.18	
2017	17.62	6.89	30.15	14.83	3.38	
2016	16.98	7.18	29.06	14.87	3.51	
2015	16.31	7.72	29.36	14.75	3.61	
2014	15.73	8.37	28.55	14.62	3.78	
2013	15.3	8.47	27.76	14.43	3.79	
2012	14.92	8.71	27.01	14.44	3.89	
2011	14.69	8.94	26.19	14.59	3.94	
2010	14.5	9.23	25.25	14.91	3.99	
2009	14.23	9.39	24.13	15.43	4.12	

*Education: Low = high school or less, Moderate = some college or associate degree, High = bachelor’s degree or higher. Race: 1 = White, 2 = Black, 3 = American Indian/Alaska Native, 4 = Asian or Pacific Islander.*

In 2022, adverse birth outcomes varied by maternal and birth characteristics. Younger mothers (<25 years) had the highest prevalence (17.6%), followed by older mothers (>34 years, 15.0%). Mothers who smoked experienced substantially higher ABOs (25.1%) compared with non-smokers (15.1%), and inadequate prenatal care was associated with nearly double the risk (29.9% vs. 14.3% for adequate care). The presence of pregnancy-related predictive variables increased ABO prevalence to 21.1% compared with 12.5% among mothers without such risks. Short birth intervals (<2 years) were also associated with higher ABOs (20.0% vs. 14.2%). ABO prevalence decreased with increasing maternal education (low: 18.0%, moderate: 15.8%, high: 12.4%) and was higher among unmarried mothers (19.1%) than married mothers (13.6%). Racial disparities were evident, with Black mothers having the highest ABOs (22.3%), followed by AIAN (17.8%), Asian/Pacific Islander (15.5%), and White mothers (14.0%) (Table 2, S2 Text).

**Table 2:** Model compression results and RMSE-based variable contribution analyses for predicting adverse birth outcomes.

**I. For model compression**				
**Model type**	**Coefficient**		**RMSE**	
AR(1)	0.72	0.266		
AR12		0.274		
Auto		0.274		
Deep		0.181		
**II. RMSE-based assessment of variable contribution to adverse birth outcome prediction in the US**				
Include all independent variables.		0.3816	0.28	0.233
Drop Pregnancy risk		0.37	0.28	0.32
Drop BMI		0.28	Exclude	Exclude
Drop maternal age		0.33	0.36	0.29
Drop Cigarette smoking		0.36	0.36	0.27
Drop prenatal care		0.387	0.23	Exclude
Drop birth interval		0.444	0.309	0.38
**II. Measuring the contribution to prediction performance on adverse birth outcomes by maternal BMI**				
Low		1.13		
Normal+		0.273		
**II. Measuring the contribution to prediction performance on adverse birth outcomes by maternal Education**				
Low		0.527		
Moderate		0.384		
High		0.263		
**II. Measuring the contribution to prediction performance on adverse birth outcomes by marital status**				
Married		0.216		
Single		0.528		
**II. Measuring the contribution to prediction performance on adverse birth outcomes by prenatal care**				
Inadequate		0.679		
Adequate		0.197		
**II. Measuring the contribution to prediction performance on adverse birth outcomes by race**				
White		0.243		
Black		0.387		
AIAN		0.801		
Asian or Pacific Islander		0.477		

### Temporal trends in adverse birth outcomes

Time-series analysis using the MLP model showed a gradual increase in overall adverse birth outcomes during the observation period, with model-based projections indicating continued increases through the forecast horizon. Preterm birth and low birth weight were the primary contributors to overall adverse birth outcomes, whereas low 5-minute Apgar scores and neonatal mortality contributed comparatively smaller effects. Preterm birth exhibited long-term cyclical patterns, suggesting the contributing patterns of persistent structural and environmental drivers (Figs 1A and S7).

Pregnancy-related medical conditions and advanced maternal age (>34 years) showed the largest predictive contributions to model performance and exhibited progressive upward trends over time. Short birth intervals (<2 years) were consistently associated with higher predicted ABO rates. In contrast, maternal smoking prevalence declined substantially, resulting in reduced predicted impacts on future ABO rates (Figs 1B and S8).

### Racial and ethnic disparities

Marked disparities in ABOs were observed across racial and ethnic groups. Black mothers consistently experienced the highest burden of adverse birth outcomes across both observed and forecasted periods. American Indian/Alaska Native mothers demonstrated variable temporal patterns, with declining adverse birth outcome rates during the observation period but model-projected increases in neonatal mortality toward the end of the forecast horizon. Potentially associated with increasing prevalence of pregnancy-related medical conditions over time (Figs 2 and S9).

White mothers exhibited an initial increase in ABOs followed by a projected decline, while Asian/Pacific Islander mothers consistently experienced the lowest ABO rates, although their relative improvement was smaller compared with White and AIAN populations. Notably, projected child mortality declined most substantially among Black mothers, whereas AIAN child mortality was projected to potentially exceed that of all other groups by the end of the forecast period (Figs 2 and S9).

### Educational disparities

Mothers with lower educational attainment experienced the highest prevalence of ABOs, with disparities widening between 2009 and 2023. Although low birth weight increased across all education levels, it remained most pronounced among lower-educated mothers, who also exhibited the highest rates of preterm birth and infant mortality (Figs 3 and S10).

During the forecast period, mortality trajectories diverged by education level: mortality declined among higher educated mothers but rebounded among lower educated mothers. Preterm birth rates continued to increase most rapidly in the lower education group, while stabilizing or declining among mothers with higher education. These disparities were observed alongside increasing prevalence of pregnancy-related risk factors and persistently shorter birth intervals among lower-educated mothers (Figs 3 and S10).

### Prenatal care adequacy

Adequate prenatal care (PNC) utilization varied by maternal age, with older mothers more likely to receive adequate care. Smoking prevalence declined most sharply among mothers receiving adequate PNC; however, these mothers also exhibited higher pregnancy-related risks, reflecting targeted care among high-risk pregnancies. Mothers receiving inadequate PNC were more likely to have short birth intervals (<2 years), which contributed to elevated ABO risks (Figs 4 and S11).

### Maternal BMI and marital status

Underweight mothers (BMI < 18.5) experienced substantial projected increases in multiple ABO outcomes, particularly during the forecast period (Figs 5 and S12). Marital status analyses indicated increasing ABO trends among married mothers, while unmarried mothers exhibited declining trends during most of the observation and forecast periods; however, this pattern reversed in the final forecast year (S13 and S14 Figs)

### Predictor contributions and model performance

The MLP model identified pregnancy-related medical conditions and advanced maternal age (>34 years) as the most influential predictors of adverse birth outcomes, corresponding to prevalence’s of 35.39% and 20.91% in 2023, respectively. Additional contributors included low maternal BMI (<18.5%), lower educational attainment, and short birth intervals (<2 years).

The model achieved an RMSE of 0.181 for predicting overall adverse birth outcomes at the population level. To put this in plain terms: the ABO rate stayed between 14.9 and 15.5 from 2009 to 2023, so an RMSE of 0.181 means the model’s typical error is about 0.18 percentage points, for instance, guessing 15.3% when the true rate is 15.5%. Predictive performance was reduced in certain subgroups, including American Indian/Alaska Native mothers, underweight mothers, and mothers receiving inadequate prenatal care, reflecting increased uncertainty in smaller or higher-risk populations.

Seasonal variation in adverse birth outcomes was observed, with higher rates during winter months across the study period.

## Discussion

The identification of pregnancy-related medical conditions and advanced maternal age as variables with the largest predictive contribution highlights the increasing prevalence of high-risk pregnancy characteristics in the United States. Reduced predictive accuracy among AIAN mothers, underweight mothers, and those receiving inadequate prenatal care likely reflects structural inequities, smaller subgroup sizes, and greater heterogeneity in risk profiles. This study was designed for population-level forecasting and predictive modeling rather than causal inference. The model is meant for forecasting and risk stratification, not for explaining why outcomes occur. Accordingly, the reported subgroup patterns should be interpreted as population-level predictive signals rather than causal effects or individualized clinical risk estimates [18].

The observed seasonal peaks in adverse birth outcomes, combined with projected increases among AIAN and underweight populations through 2030, suggest that targeted interventions addressing maternal nutrition, prenatal care access, and optimal birth spacing may be particularly impactful in mitigating future disparities.

This study demonstrates the utility of time-series MLP neural networks in predicting adverse birth outcomes (ABOs) and identifying vulnerable groups, flagging at-risk subpopulations, and highlighting patterns that may not be obvious from a simple trend line in the United States. Over the 2009–2023 period, ABO prevalence increased slightly, with persistent disparities by race, maternal BMI, education, and prenatal care access. Black and AIAN mothers consistently experienced the highest ABO rates, reflecting longstanding structural inequities in healthcare and social associated characteristics of health [1,4,9].

The MLP model identified pregnancy-related medical complications and advanced maternal age (>34 years) as the strongest predictive contributions of ABOs. Underweight mothers and those with lower educational attainment faced disproportionately high risks of preterm birth, low birth weight, and neonatal mortality. Short interpregnancy intervals and inadequate prenatal care further compounded these risks. These findings highlight complex associations among biological, social, and structural characteristics captured by the predictive model, which conventional epidemiological approaches may not fully capture [1,4,19].

Time-series analysis revealed seasonal patterns, with winter months associated with higher ABO rates, likely due to environmental stressors, infectious disease prevalence, and temperature-related effects on fetal growth. Forecasts to 2030 indicate worsening outcomes for AIAN mothers and underweight populations, underscoring the need for targeted interventions [4,20–22].

The predictive performance of the MLP model may support public health surveillance and maternal health planning. The forecasts may help identify populations experiencing elevated risk and inform allocation of maternal health resources at the population level [23]. Furthermore, integrating ML-driven forecasts into public health strategies supports data-driven decision-making aligned with Sustainable Development Goal 3 [24–27]. Early work on technology-enabled intensive care models has emphasized the importance of integrated data systems, decision support, and continuous performance monitoring as foundations for improving clinical outcomes and standardizing care processes [28].

This study highlights the value of predictive modeling for improving maternal and neonatal health. Persistent racial and socioeconomic disparities in adverse birth outcomes indicate the need for targeted, equity-focused interventions. Key modifiable predictive variables include pregnancy-related complications, advanced maternal age, underweight status, inadequate prenatal care, and low educational attainment. Machine learning models may support population-level risk surveillance and maternal health resource planning, while observed seasonal patterns suggest opportunities for time-sensitive preventive strategies. Because the analysis was based on aggregated observational data, the findings should be interpreted as predictive associations rather than causal relationships. Formal uncertainty quantification methods, including bootstrap-based prediction intervals or Bayesian forecasting approaches, were not implemented in the current study.

## Limitations

Several limitations must be considered. First, ABOs were defined using four indicators (preterm birth, low birth weight, low Apgar score, and neonatal mortality), which may not fully capture all adverse pregnancy outcomes. Second, the study period (2009–2023) may limit the assessment of longer-term trends and continuous patterns. Third, while the MLP model demonstrated strong predictive performance, it may not fully capture all complex relationships among variables, particularly for small subgroups such as AIAN mothers and underweight women. Model forecasts depend on historical data trends and may be affected by unexpected events such as global health crises or policy changes. Certain confounders, including genetic predispositions and environmental exposures, were not included due to data limitations. Finally, some high-risk populations were underrepresented in the dataset, potentially affecting the generalizability of predictions. The validation strategy used only a single holdout year (2023) for testing. A rolling origin or time series cross-validation would give a more robust assessment of forecast stability; future work should address this.

Formal prediction intervals or uncertainty bands (e.g., bootstrap-based 95% prediction intervals) were not estimated for forecasts through 2030 and therefore forecast uncertainty may be underestimated, particularly for smaller subgroups. The supplementary figures show some forecast variability across model runs, but proper uncertainty measures (e.g., 95% bootstrap intervals) are not provided. Forecasts for smaller subgroups, particularly American Indian/Alaska Native mothers and underweight mothers, should therefore be interpreted cautiously. Forecast trajectories for small subgroups (AIAN, underweight) show abrupt shifts near the forecast horizon, likely due to data scarcity and extrapolation artifacts rather than true future signals. The composite ABO rate is 15%, so class imbalance was not severe; no rebalancing techniques were applied.

The analysis uses aggregated rates (national and subgroup levels), so the findings are ecological and may not translate to individual patients. The COVID-19 years (2020–2021) may have caused sudden shifts in birth outcomes and healthcare access; the model was not specifically tested for such structural breaks. There is no external validation on data from other countries or different time periods. Finally, the forecasts are intended for population-level surveillance and planning rather than individual clinical decision-making. Using these predictions at the patient level would be inappropriate.

## Conclusion

Time-series multilayer perceptron neural networks provide a useful approach for forecasting adverse birth outcomes and identifying populations experiencing elevated risk in the United States. By leveraging longitudinal vital statistics data, this study highlights key predictive factors of ABOs, including pregnancy-related complications, advanced maternal age, low BMI, inadequate prenatal care, and low education. Structural inequities contribute to persistent racial disparities, emphasizing the need for targeted, equity-focused interventions.

Future research should incorporate additional predictors, extend analysis over longer timeframes, and evaluate model performance across diverse populations. Predictive modeling approaches may support maternal health surveillance, resource allocation, and population-level public health planning aimed at reducing disparities in maternal and neonatal outcomes.

## Supporting information

S1 Fig Decomposition of adverse birth outcomes using additive time series: trends, seasonal patterns, and residual fluctuations. (PNG)

S2 Fig Figure showed ACF plot of birth outcomes of temporal dependencies and seasonal patterns. (PNG)

S3 Fig MLP model description and architecture diagram of the overall adverse birth outcome. (PNG)

S4 Fig MLP model description and architecture diagram of the indicators and factors including demographic and pregnant related risk groups. (PNG)

S5 Fig MLP model predictions for adverse birth outcome and its key components in the last 12 months (2023). (PNG)

S6 Fig MLP model predictions for adverse birth outcome influenced factors in the Last 12 Months (2023). (PNG)

S7 Fig MLP model predictions for adverse birth outcome and its key components in the next 84 months (2023). (PNG)

S8 Fig MLP model predictions for adverse birth outcome influenced factors in the Last 12 Months (2023). (PNG)

S9 Fig Trends and predictions of maternal health factors by race. (PNG)

S10 Fig Trends and predictions of maternal health factors by education. (PNG)

S11 Fig Trends and predictions of maternal health factors by prenatal care status. (PNG)

S12 Fig Trends and predictions of maternal health factors by BMI. (PNG)

S13 Fig Trends and predictions of adverse birth outcomes by marital status. (PNG)

S14 Fig Trends and predictions of maternal health factors by marital status. (PNG)

S1 Text Extended methods. Detailed description of variable categorization, model evaluation, time-series decomposition, lag selection, multilayer perceptron architecture, model training, forecasting procedures, and interpretation of supplementary figures.
(DOCX)

S2 Text Extended results. Supplementary results describing temporal trends, autocorrelation and seasonality analyses, subgroup-specific forecasts of adverse birth outcomes and risk factors, and predicted patterns by race/ethnicity, education, prenatal care status, BMI, and marital status.
(DOCX)

S1 Table Descriptive statistics of adverse birth outcomes by maternal risk factors and demographic characteristics. Annual percentages of adverse birth outcomes from 2009–2023 stratified by maternal age, cigarette smoking, prenatal care adequacy, body mass index (BMI), pregnancy-related risk factors, birth interval, education level, marital status, and race/ethnicity.
(DOCX)
