# Supplementary figures and images for: Time-series prediction of adverse birth outcomes in the U.S. using multilayer perceptron neural networks
Source: PLOS Digit Health. 2026 Jul 1;5(7):e0001515. doi: 10.1371/journal.pdig.0001515 (PMC13322551; doi:10.1371/journal.pdig.0001515)

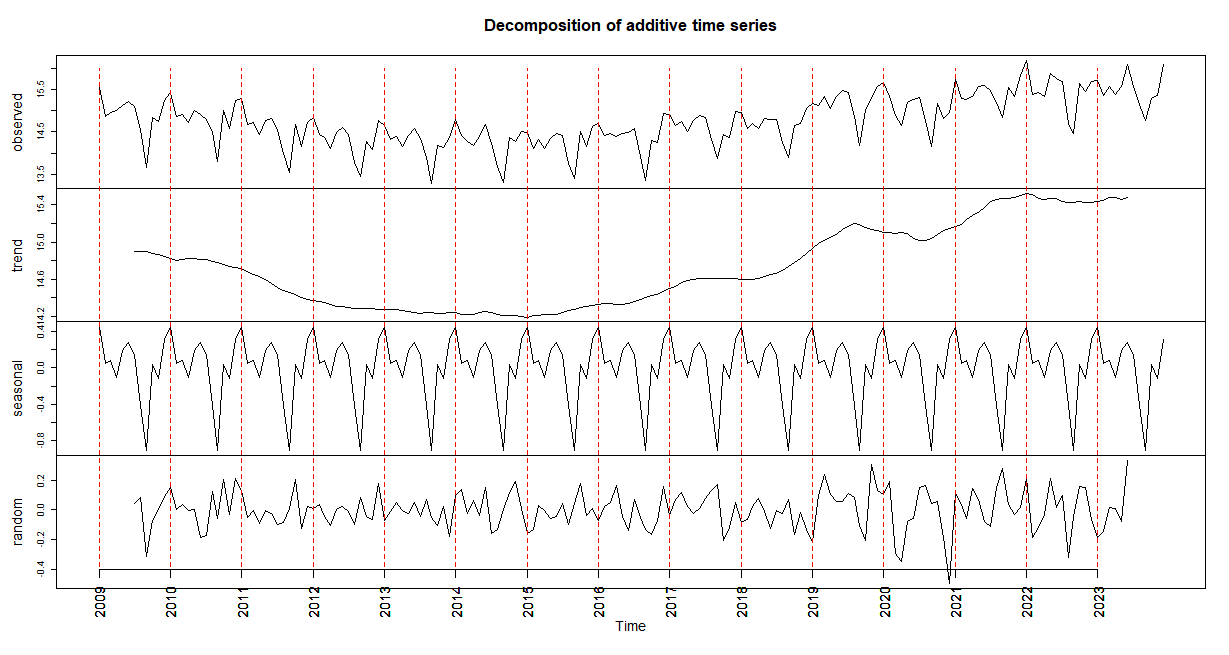

Supplement: S1 Fig — (PNG) [file pdig.0001515.s001.png]

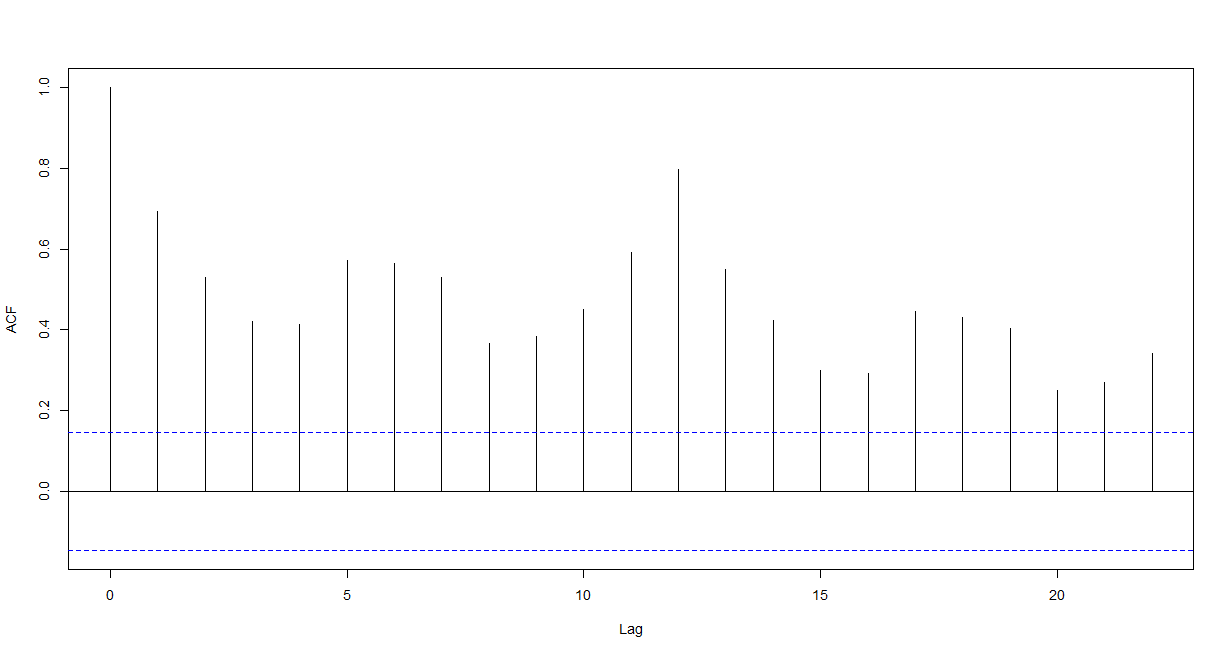

Supplement: S2 Fig — (PNG) [file pdig.0001515.s002.png]

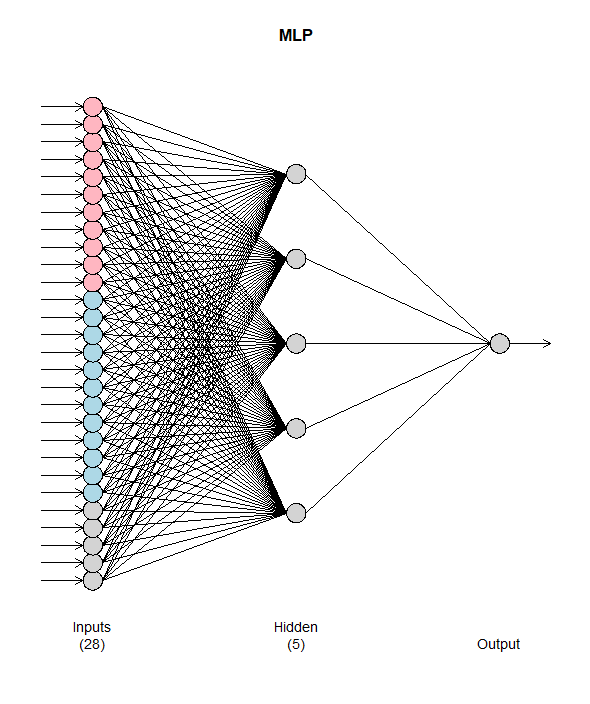

Supplement: S3 Fig — (PNG) [file pdig.0001515.s003.png]

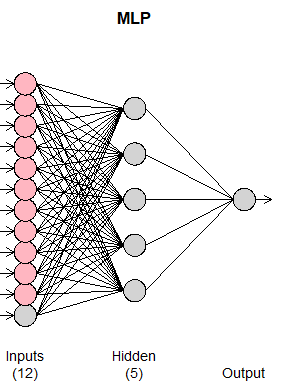

Supplement: S4 Fig — (PNG) [file pdig.0001515.s004.png]

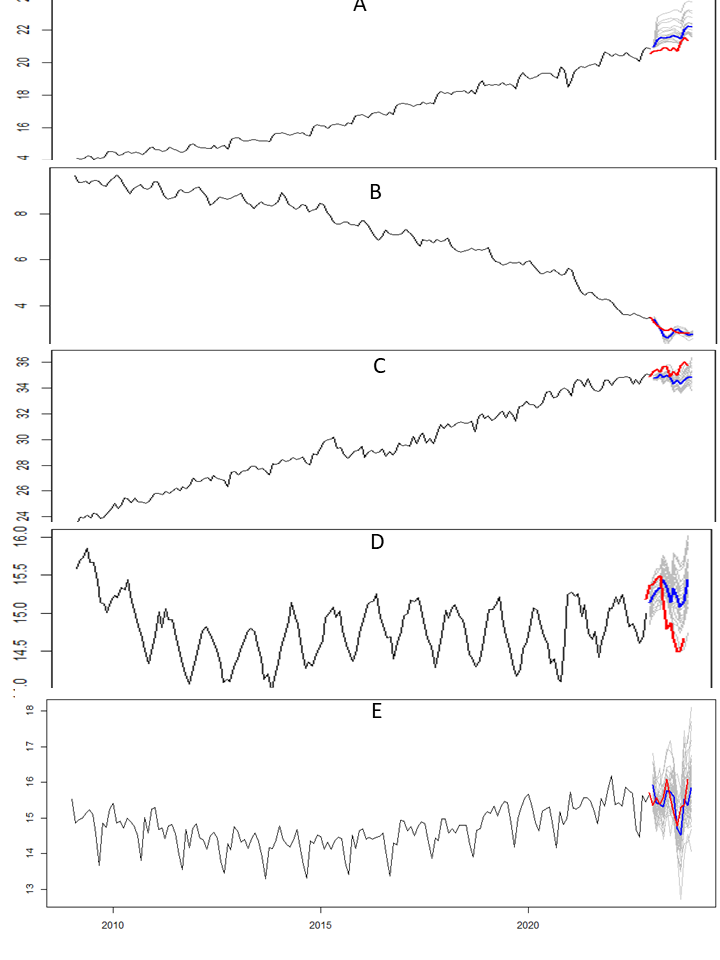

Supplement: S5 Fig — (PNG) [file pdig.0001515.s005.png]

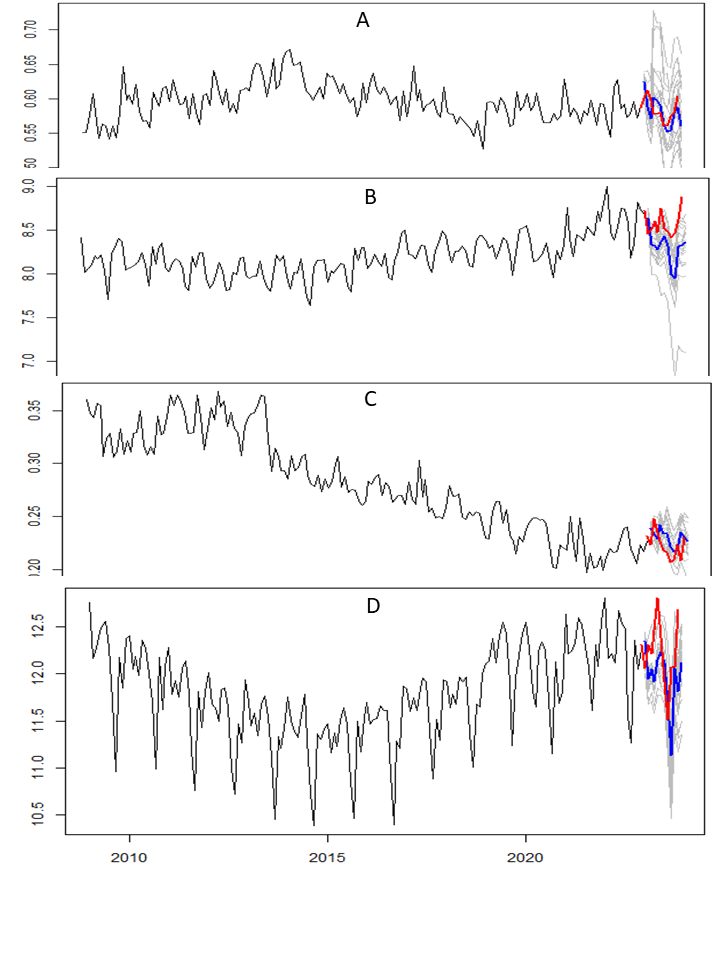

Supplement: S6 Fig — (PNG) [file pdig.0001515.s006.png]

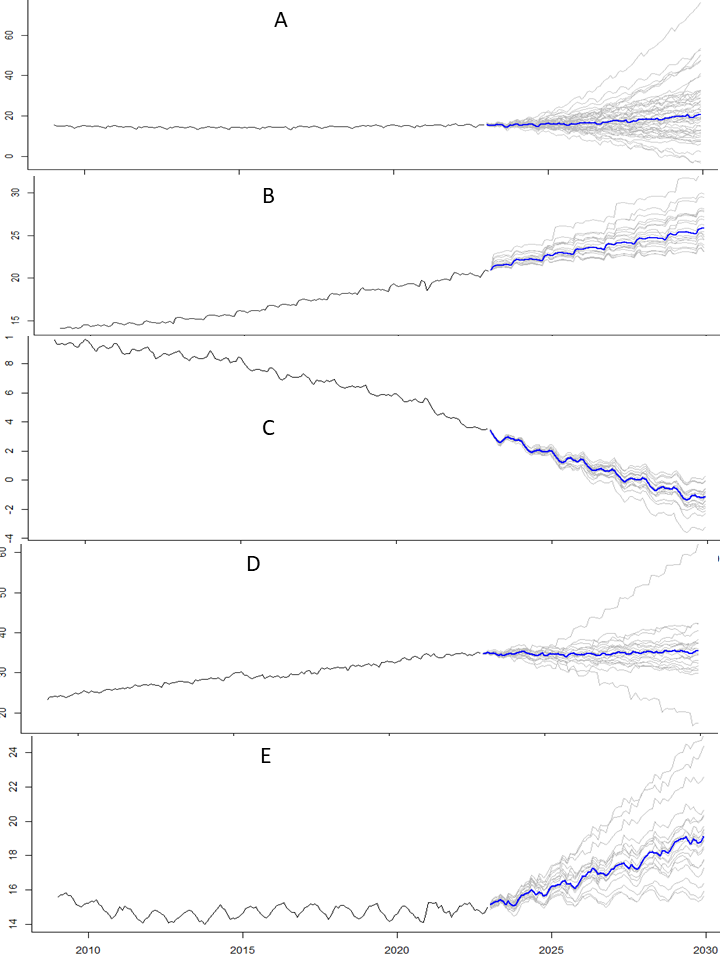

Supplement: S7 Fig — (PNG) [file pdig.0001515.s007.png]

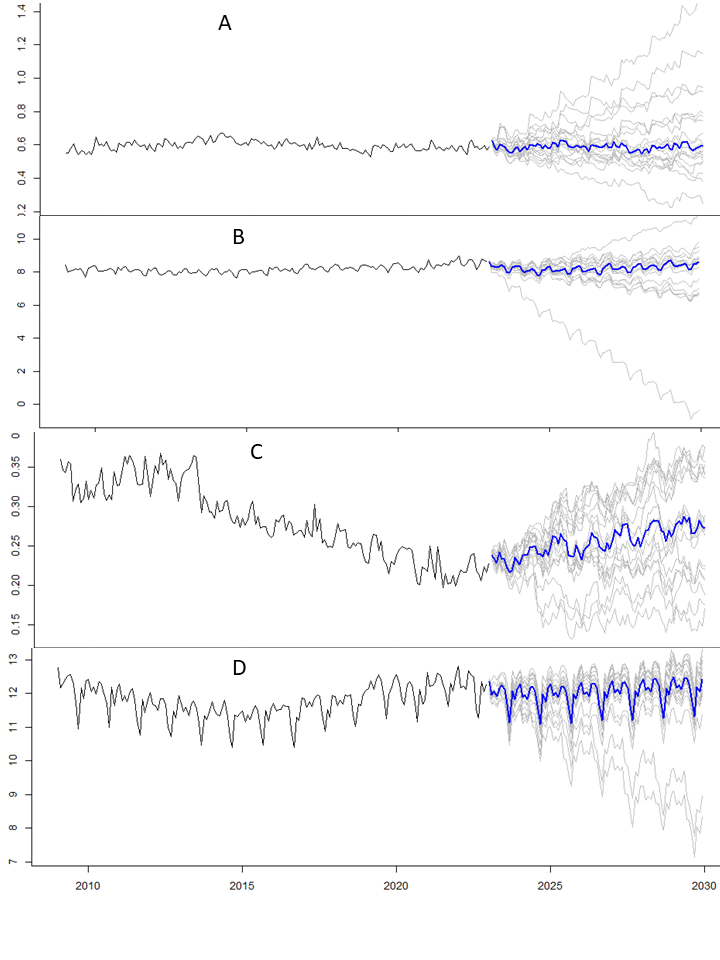

Supplement: S8 Fig — (PNG) [file pdig.0001515.s008.png]

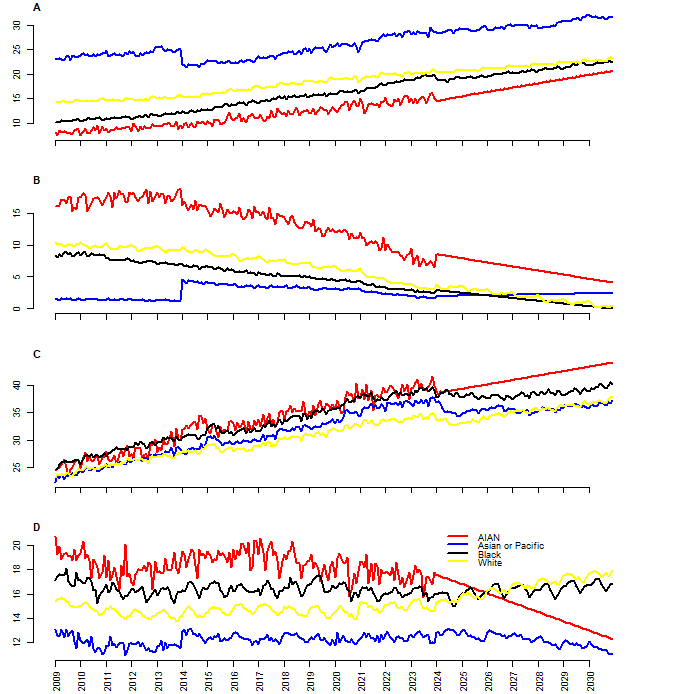

Supplement: S9 Fig — (PNG) [file pdig.0001515.s009.png]

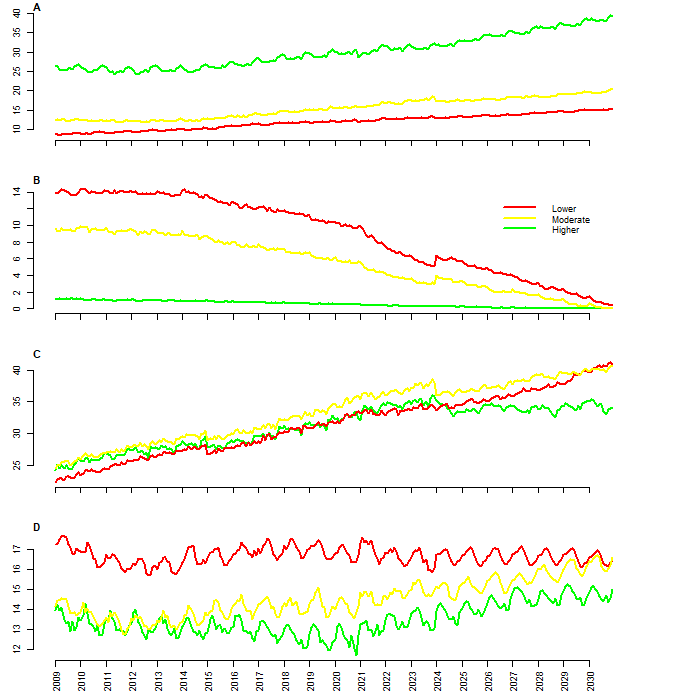

Supplement: S10 Fig — (PNG) [file pdig.0001515.s010.png]

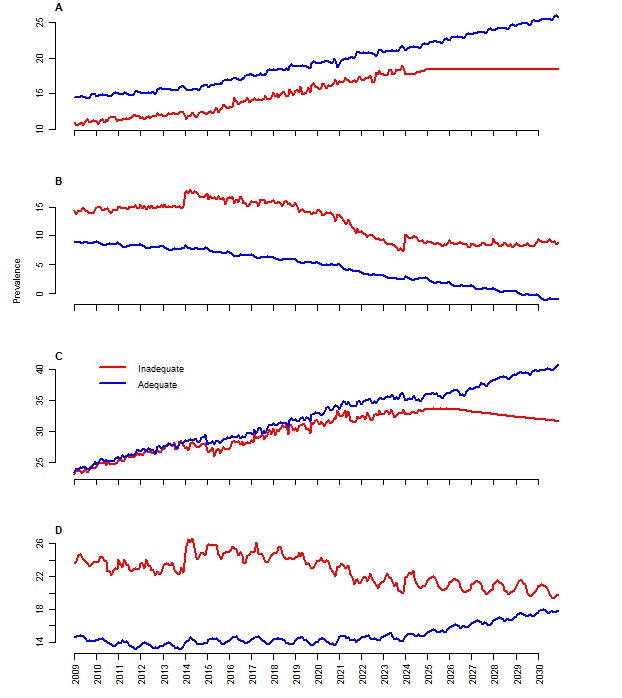

Supplement: S11 Fig — (PNG) [file pdig.0001515.s011.png]

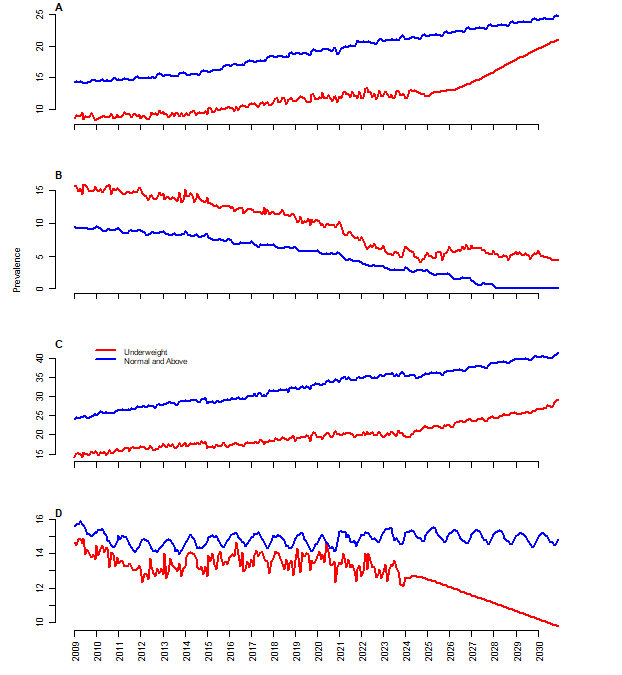

Supplement: S12 Fig — (PNG) [file pdig.0001515.s012.png]

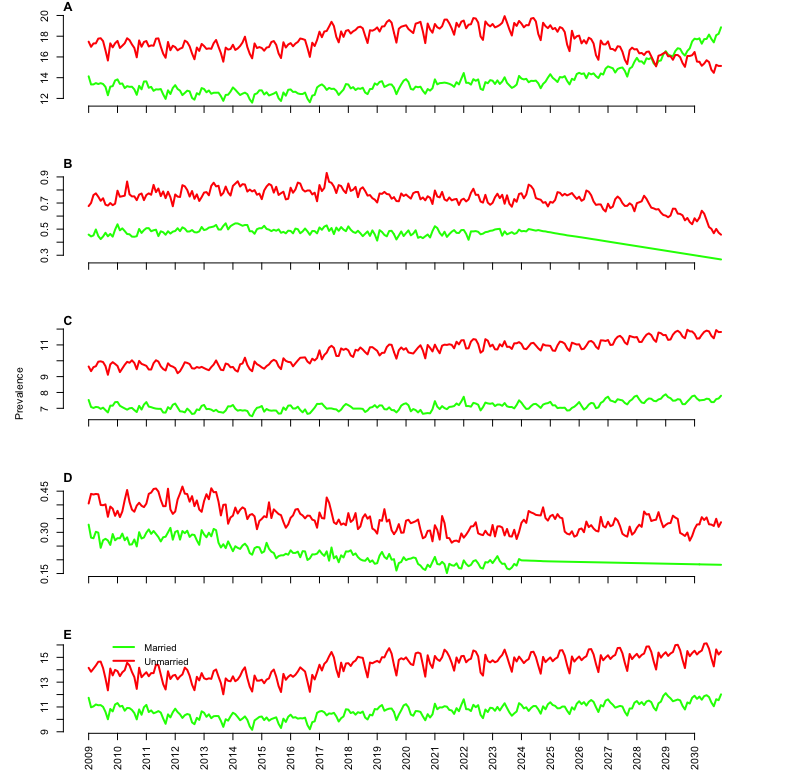

Supplement: S13 Fig — (PNG) [file pdig.0001515.s013.png]

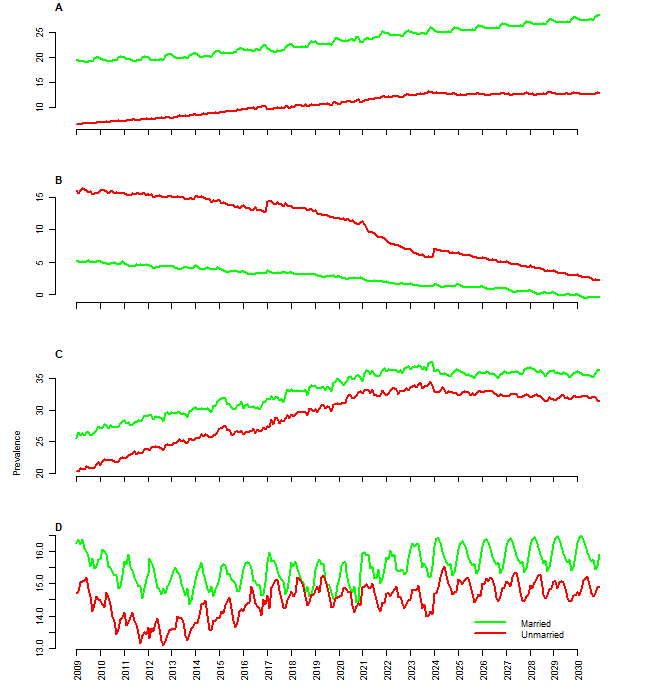

Supplement: S14 Fig — (PNG) [file pdig.0001515.s014.png]
