# Supplementary material for: Time-series prediction of adverse birth outcomes in the U.S. using multilayer perceptron neural networks
Source: PLOS Digit Health. 2026 Jul 1;5(7):e0001515. doi: 10.1371/journal.pdig.0001515 (PMC13322551; doi:10.1371/journal.pdig.0001515)
Supplement: S1 Text — Detailed description of variable categorization, model evaluation, time-series decomposition, lag selection, multilayer perceptron architecture, model training, forecasting procedures, and interpretation of supplementary figures. (DOCX) [file pdig.0001515.s015.docx]

# S1 Text Extended Methods

## Categorization and handling of variables for predicting adverse birth outcomes:

The primary outcome was a composite ABO, coded as 1 if any of the following occurred: preterm birth (<37 weeks), low birth weight (<2.5 kg), 5-min Apgar <4, or neonatal death; otherwise coded as 0.

**Predictor Variables**

Independent variables used for prediction included:

- **Maternal age:** <35 years (0), ≥35 years (1)
- **Cigarette smoking during pregnancy:** no (0), yes (1)
- **Pregnancy-related medical risk:** absent (0), present (1)
- **Birth interval:** ≥2 years (0), <2 years (1)

**Stratification Variables**

Analyses were stratified to assess heterogeneity across subgroups by prenatal care adequacy, maternal BMI, marital status, education, and race/ethnicity.

- **Prenatal care adequacy:** ≥5 visits (adequate = 0), <5 visits (inadequate = 1)
- **Maternal BMI:** ≥18.5 kg/m² (0), <18.5 kg/m² (1)
- **Marital status:** married (0), unmarried (1)
- **Education:** low (≤high school/GED = 0), moderate (some college/associate degree = 1), high (bachelor’s degree or higher = 2)
- **Race/ethnicity:** White (1), Black (2), American Indian/Alaska Native (3), Asian/Pacific Islander or other/multiple race (4)

## ****Model Evaluation and Variable Selection****

Temporal dependence was assessed using an autoregressive neural network equivalent to AR(1). As shown in, the estimated AR(1) coefficient was **0.72**, indicating moderate temporal persistence in adverse birth outcomes across consecutive years. This supports the inclusion of lagged outcome information in time-series prediction.

Variable contribution was evaluated using RMSE-based drop-one-variable-at-a-time analyses. The full model RMSE was 0.3816 (Including all covariates). Interestingly, exclusion of maternal BMI and prenatal care slightly reduced RMSE to 0.233, suggesting lower incremental contribution within the multivariable MLP framework, possibly due to multicollinearity. Removal of other predictors increased RMSE, reflecting higher predictive importance.

## Time-Series Decomposition and Lag Selection

Additive time-series decomposition was applied to separate long-term trends, seasonal patterns, and residual variation in adverse birth outcomes (**S1 Fig**). Autocorrelation analysis demonstrated a clear seasonal pattern, with the highest correlation observed at a **12-month lag** (**S2 Fig**). Accordingly, a lag of 12 months was consistently applied across all MLP models. From 2009 to 2015, ABOs declined, then gradually increased, reaching a peak of 15.5% in 2022. This pattern largely reflects trends in preterm birth and low birth weight, whereas 5-minute Apgar scores and neonatal survival showed different trends and contributed less to the overall ABO pattern.

The ACF plot represents the correlation between birth outcomes across different time lags. The Fig showed a wave-like pattern (down-up-down-up) in the ACF plot indicating a cyclical or seasonal trend in adverse birth outcomes. The autocorrelation function (ACF) indicated significant correlation at a lag of 12 months, which was used consistently across all MLP models. Lag-12 exhibited the highest autocorrelation and was therefore used as the default input lag for all MLP models, both overall and in subgroup analyses.

## ****Multilayer Perceptron Architecture****

The MLP model included input nodes representing lagged outcomes and four primary predictors, processed through five hidden layers, with a single output layer predicting ABOs. Models were trained using backpropagation with 50 repetitions. The number of input nodes varied slightly in subgroup analyses based on available predictors (S3 Fig). Group-specific architectures for outcome components and predictor sets are illustrated in **S4 Fig**.

The MLP model for adverse birth outcomes overall and in each group is designed with different default input nodes representing four independent variables, processed through five hidden layers to capture complex patterns and relationships in the data with 50 training repetitions. The number of input nodes varied by subgroup, reflecting differences in available predictors across stratified analyses (S4 Fig).

## ****Model Training and Forecasting****

The first **168 months** of data were used for model training, and the final **12 months** were reserved for testing. Forecasts were generated for overall adverse birth outcomes, individual components, and influencing factors (**S5–S6 Figs**).

In all figures, the solid black line represents the trend learned from the training data, the red line indicates observed test data, thin black lines show forecast distributions, and the bold blue line represents the median forecast.

The MLP (Multilayer Perceptron) model is designed with a single outcome layer representing one dependent variable, optimized using activation functions and backpropagation. This architecture effectively predicts adverse birth outcomes based on the given input features.

Structured with 12 input nodes (determined by the highest ACF value), the model processes data through five hidden layers before reaching the outcome layer. The MLP captures complex interactions among the four input predictors and lagged outcomes through its hidden layers.

The accompanying diagram visualizes the MLP model’s structure, detailing its components and predictive factors for adverse birth outcomes. By employing activation functions and backpropagation, the model captures complex relationships across multiple layers. A lag of 12 is consistently applied across all cases.

S5 Fig MLP model predictions for adverse birth outcome and its key components in the last 12 months (2023). This Fig presents forecasts generated by the MLP model for adverse birth outcomes and its key components of adverse birth outcomes over the final 12 months of 2023: Using an MLP model (A- Overall, B- 5-Minute APGAR, C- Birth weight, D- Mortality, E- Preterm birth)

S6 Fig MLP model predictions for adverse birth outcome influenced factors in the Last 12 Months (2023). This Fig presents forecasts generated by the MLP model for key factors of adverse birth outcomes over the final 12 months of 2023: Childbirth at age 35 and above(A), Cigarette smoking(B), Pregnancy-related risks(C), Birth interval below two years (D)

The first 168 months of data were used for model training, and the final 12 months were reserved for testing. The test period predictions were generated based on the trained model. Adverse birth outcomes, along with their components and influencing factors, were analyzed both overall and across different maternal and demographic.

In all related Figs, the solid black line represents the trend learned from the training data, the red line depicts the actual trend from the test data, multiple black lines illustrate the forecasted values for the test period, and the bold blue line indicates the median forecast.
