# Supplementary material for: Time-series prediction of adverse birth outcomes in the U.S. using multilayer perceptron neural networks
Source: PLOS Digit Health. 2026 Jul 1;5(7):e0001515. doi: 10.1371/journal.pdig.0001515 (PMC13322551; doi:10.1371/journal.pdig.0001515)
Supplement: S2 Text — Supplementary results describing temporal trends, autocorrelation and seasonality analyses, subgroup-specific forecasts of adverse birth outcomes and risk factors, and predicted patterns by race/ethnicity, education, prenatal care status, BMI, and marital status. (DOCX) [file pdig.0001515.s016.docx]

# S2 Text Extended Result

From 2009 to 2015, ABOs declined, then gradually increased, reaching a peak of 15.5% in 2022. This pattern largely reflects trends in preterm birth and low birth weight, whereas 5-minute Apgar scores and neonatal survival showed different trends and contributed less to the overall ABO pattern.

The ACF plot represents the correlation between birth outcomes across different time lags. The Fig showed a wave-like pattern (down-up-down-up) in the ACF plot indicating a cyclical or seasonal trend in adverse birth outcomes. The autocorrelation function (ACF) indicated significant correlation at a lag of 12 months, which was used consistently across all MLP models. Lag-12 exhibited the highest autocorrelation and was therefore used as the default input lag for all MLP models, both overall and in subgroup analyses.

The seasonality indicated that the adverse birth outcome has regular periodic variations in the periods of months. The seasonal occurrence at the month of the start and end of each year showed the highest occurrence. This indicates that the winter season is more risky. Additive time-series decomposition was used to separate long-term trends, seasonal patterns, and residual components of adverse birth outcomes (S1 Fig). The second peak occurred in the summer, which may be explained by the higher rate of preterm births observed each year.

## Descriptive analysis of adverse birth outcomes by maternal and birth characteristics 2009—2023

In the year 2022 from mothers aged at this birth above 34 years old 14.96% and below 25 years old 17.57% of them suffering adverse birth outcomes. From non-cigarette smoking 15.07% and cigarette smoking 25.05% suffered adverse birth outcomes. Mothers with adequate prenatal care were 14.34% and from inadequate prenatal care, 29.89% of them suffered adverse birth outcomes. Of newborn mothers without pregnancy risk factors 12.46% and with pregnancy risk factors 21.05% of them were suffering adverse birth outcomes. Mothers born within two years of interval 20.02% and above two years of interval 14.2% of they were suffering adverse birth outcomes. In the year 2022 new birth mothers whose education is lower 18.01%, moderate 15.76%, and higher 12.43% of them suffering adverse birth outcomes. 13.55% of married women and 19.05% of unmarried women were suffering adverse birth outcomes. Additionally, 13.97% of whites, 22.33% of blacks, 17.81% of AIAN, and 15.45% of Asian or Pacific Islanders suffered from adverse birth outcomes (S1 Table).

## Forecasting the next 84 months of adverse birth outcome components and factors

S7 Fig. MLP model predictions for adverse birth outcome and its key components in the next 84 months (2023): This Fig presents forecasts generated by the MLP model for adverse birth outcomes and its key components of adverse birth outcomes over the next 84 months (2024—2030): Using an MLP model (A- Overall, B- 5-Minute APGAR, C- Birth weight, D- Mortality, E- Preterm birth)

S8 Fig MLP model predictions for adverse birth outcome influenced factors in the Last 12 Months (2023). This Fig presents forecasts generated by the MLP model for key factors of adverse birth outcomes over the final 12 months of 2023: Childbirth at age 35 and above(A), Cigarette smoking(B), Pregnancy-related risks(C), Birth interval below two years (D)

The overall adverse birth outcomes, along with each component and contributing factor, are predicted over the next 84 months. Additionally, predictions for each component and factor are made separately for each pregnancy and socio-demographic group. In all related Figs, the solid black line represents the trend learned from the training data, multiple black lines illustrate the forecasted values for the prediction period, and the bold blue line indicates the median forecast.

S9 Fig Trends and predictions of maternal health factors by race. Childbirth at age 35 and above(A), Cigarette smoking(B), Pregnancy-related risks(C), Birth interval below two years (D)

In terms of **age at childbirth**, Asian and Pacific Islander races exhibited the highest contribution to childbirth among mothers above 34 years of age. White mothers followed as the second highest contributors, with Black mothers being the third. Throughout the study period, the trend showed that all racial groups increased their contributions over time. Initially, there was a significant gap between the racial groups, but this gap narrowed as the study progressed. Black and White mothers showed a similar rate towards the end of the study period, with the AIAN group approaching them. However, the Asian and Pacific Islander groups maintained a larger gap compared to the other racial groups, continuing to contribute at a higher rate in older maternal age.

In terms of **cigarette smoking** among mothers, all racial groups exhibited a decline over time, both in the study and prediction periods. Black and White mothers showed a sharp reduction in smoking rates, while the Asian and Pacific Islander group, which initially had the lowest smoking rates, saw a smaller decline during the prediction period. In the final year of the prediction period, Black and White mothers approached the null point, whereas the Asian and Pacific Islander and AIAN groups continued to have higher smoking rates.

Regarding **pregnancy risk**, all racial groups showed an increase in pregnancy-related risks during both the study and prediction periods. At the start of the study, there was little difference between the groups, but over time, gaps began to emerge. The AIAN group registered the highest pregnancy risk, followed by Black mothers. All races showed significantly high pregnancy-related risks, and no racial group showed a decrease in risk over time.

In terms of the **birth interval**, there was initially a wider gap between the groups, with the AIAN group showing the highest contribution and more fluctuation. The AIAN group also showed a reduction in birth interval during the study period and a sharp reduction in the prediction period, causing the group to shift from the highest to the third highest contributor for births with intervals below 2 years. Black mothers contributed the second highest in the beginning, but as White mothers showed an increasing trend over time, the ranking shifted, with White mothers moving from third to second, and Black mothers moving to third. Asian and Pacific Islander mothers initially had the lowest contribution but showed a consistent trend, with a reduction in the last prediction period, although they still had the lowest birth interval.

S10 Fig Trends and predictions of maternal health factors by education: Childbirth at age 35 and above(A), Cigarette smoking(B), Pregnancy-related risks(C), Birth interval below two years (D). Mothers with higher education had a higher proportion of childbirth at age 34 and above, while those with lower and moderate education had lower rates in comparison. Over time, the trend indicates that highly educated mothers increasingly delay childbirth to an older age. Both lower-and moderately-educated mothers showed a gradual increase in older-age childbirth during the study and prediction periods. Overall, less educated mothers had the lowest rates of childbirth at an older age compared to moderate and highly educated mothers.

At the beginning of the study period, lower- and moderately educated mothers had a higher proportion of cigarette smoking, whereas highly educated mothers had a lower proportion, with smoking rates being the highest among the lower-educated group. Over time, cigarette smoking declined across all education levels. By the end of the prediction period, smoking rates among mothers are expected to approach near zero.

Pregnancy-related risks were high across all education groups, with a substantial and concerning increase during both the study and prediction periods. Over an extended period, moderately educated mothers had the highest rates of pregnancy-related risks. However, by the end of the prediction period, lower-educated mothers emerged as the most affected group, with only a small difference from the moderate-education group. Mothers with higher education initially showed an increasing trend similar to the other groups, but at the beginning of the prediction period, their risk declined slightly before rising again at a slower pace. Despite this, pregnancy-related risks remained high for all education groups.

Lower educated mothers had the highest rates of childbirth within a birth interval of less than two years. Over time, their trend remained relatively unchanged. However, for moderate and highly educated mothers, the rates gradually increased. By the final prediction period, the birth interval rates among moderately educated mothers were projected to converge with those of lower-educated mothers. In general, the final prediction suggests an overall increase in the proportion of births occurring within short intervals across all education groups.

S11 Fig Trends and predictions of maternal health factors by prenatal care status. Childbirth at age 35 and above(A), Cigarette smoking(B), Pregnancy-related risks(C), Birth interval below two years (D). The study found that inadequate prenatal care (PNC) significantly contributed to adverse birth outcomes, with a more substantial impact compared to adequate PNC. Over time, both groups showed a slight increase in adverse outcomes, but this trend was more pronounced in the inadequate PNC group. The gap was particularly notable in 5-minute Apgar scores, birth weight, child mortality, and preterm births. Specifically, inadequate PNC was linked to higher rates of adverse birth outcomes across these categories, with child mortality being notably higher in the inadequate PNC group, which fluctuated but remained high by the end of the prediction period. Preterm birth rates also displayed a persistent gap between the two groups, with inadequate PNC contributing significantly more to preterm deliveries.

Underweight mothers who have a BMI less than 18.5 experienced great increases in multiple ABOs outcomes during the prediction period. If future trends continue the preterm birth rates together with birth weight rates will increase rapidly among this population whereas the five-minute Apgar scores and neonatal mortality indicate worsening conditions during the prediction interval. Increasing smoking in childbirth in older age rates is a major contributor to negative birth outcomes in underweight mothers.

S12 Fig Trends and predictions of maternal health factors by BMI Childbirth at age 35 and above(A), Cigarette smoking(B), Pregnancy-related risks(C), Birth interval below two years (D). Among mothers aged 34 and above, those with normal and above-normal BMI had higher rates compared to those with lower BMI. Over time, trends indicate that normal and above-normal BMI increased at a similar rate. However, for lower BMI, the study period showed a slower rate of increase, while the prediction period suggests a sharp rise, ultimately narrowing the gap between the two groups.

Both groups showed a significant reduction in smoking over time. Throughout all periods, underweight mothers consistently had a higher smoking rate than their counterparts. By the end of the study period and the beginning of the prediction period, underweight mothers experienced a notable decline, reducing the gap between them and their counterparts. However, over time in the prediction period, normal and above-BMI mothers showed a greater reduction in smoking, approaching near-zero levels. In contrast, smoking rates among underweight mothers increased, leading to a wider gap between the two groups.

Mothers with normal and above-normal BMI faced higher pregnancy-related risks compared to underweight mothers. Over time, both groups experienced an increasing trend in pregnancy-related risks, both in the study and prediction periods, indicating a persistent burden across all groups.

Mothers with normal and above-normal BMI had higher rates of giving birth within a birth interval of less than two years. Over time, this trend remained relatively constant with some fluctuations. In contrast, underweight mothers showed no significant change during the study period, but in the prediction period, the birth interval declined steadily, eventually approaching near-zero levels.

S13 Fig Trends and predictions of adverse birth outcomes by marital status: (A) Overall adverse birth outcomes, (B) 5-Minute Apgar score, (C) Birth weight, (D) Infant mortality, and (E) Preterm birth

The analysis showed married mothers experienced growing adverse conditions yet unmarried mothers showed decreasing trends during this study and forecast period but in the last year of the forecasting period the finding is inverse. The potential reason for this inverse in the last few years will be the higher rate of mothers older age and the lower birth interval rate increasing more in married women than unmarried women. The transition in maternal marital status did not lower preterm birth low birth weight or infant death rates among unmarried women due to the high rate of cigarette smoking.

S14 Fig Trends and predictions of maternal health factors by marital status. Childbirth at age 35 and above(A), Cigarette smoking(B), Pregnancy-related risks(C), Birth interval below two years (D). There is a significant gap in childbirth at age 34 and above between unmarried and married mothers. Married women have a higher rate of childbirth at an older age compared to unmarried mothers. This gap is expected to persist throughout the prediction period, although both groups show an increasing trend in older-age childbirth over time.

At the beginning of the study period, cigarette smoking was more prevalent among unmarried mothers compared to married mothers. Over time, both groups showed a decline in smoking rates, with unmarried mothers exhibiting a steeper reduction. By the end of the prediction period, smoking is expected to be eliminated among married mothers, whereas unmarried mothers will continue to experience smoking-related challenges.

Pregnancy-related risks were more common among married mothers than their unmarried counterparts. Both groups followed a similar trend, with a gradual increase in pregnancy-related risks from the beginning to the end of the study period. However, in the prediction period, this trend stabilizes, with minor fluctuations over time.

The rate of childbirth within a birth interval of less than two years was higher among unmarried mothers than married mothers. Both groups exhibited significant fluctuations over time. In the prediction period, the rate remains high for both groups, indicating a persistent trend of short birth intervals.
