# Supplementary material for: Time-series prediction of adverse birth outcomes in the U.S. using multilayer perceptron neural networks
Source: PLOS Digit Health. 2026 Jul 1;5(7):e0001515. doi: 10.1371/journal.pdig.0001515 (PMC13322551; doi:10.1371/journal.pdig.0001515)
Supplement: S1 Table — Annual percentages of adverse birth outcomes from 2009–2023 stratified by maternal age, cigarette smoking, prenatal care adequacy, body mass index (BMI), pregnancy-related risk factors, birth interval, education level, marital status, and race/ethnicity. (DOCX) [file pdig.0001515.s017.docx]

# S1 Table

S1 Table. Descriptive statistics of the percentage of adverse birth outcomes by maternal risk factors and demographic characteristics.

|  | | | | | | |
| --- | --- | --- | --- | --- | --- | --- |
| **I. Percentage of adverse birth outcomes by risk factors** | | | | | | |
| **Year** | **Age (<35, >35)** | **Cigarette (No, Yes)** | **Prenatal care (>5, <5)** | **BMI (Normal+, Low)** | **Pregnancy risk (No, Yes)** | **Birth interval (>2y, <2y)** |
| 2023 | 14.94, 17.57 | 15.16, 25.63 | 14.39, 30.08 | 15.26, 19.91 | 12.39, 21.11 | 14.29, 19.82 |
| 2022 | 14.96, 17.37 | 15.07, 25.05 | 14.34, 29.89 | 15.21, 20.42 | 12.46, 21.05 | 14.2, 20.02 |
| 2021 | 14.92, 17.4 | 14.96, 24.11 | 14.31, 30.44 | 15.17, 20.03 | 12.37, 21.22 | 14.16, 19.95 |
| 2020 | 14.6, 16.78 | 14.5, 23.39 | 13.93, 30.01 | 14.77, 19.54 | 12.18, 20.69 | 13.74, 19.84 |
| 2019 | 14.69, 17.14 | 14.64, 22.74 | 14.07, 30.32 | 14.89, 19.3 | 12.35, 21.06 | 13.78, 20.44 |
| 2018 | 14.22, 16.57 | 14.09, 22.35 | 13.56, 30.34 | 14.37, 18.81 | 11.97, 20.49 | 13.59, 20.22 |
| 2017 | 14.2, 16.48 | 14.02, 22.1 | 13.55, 29.78 | 14.32, 18.59 | 12.08, 20.42 | 13.55, 20.09 |
| 2016 | 13.93, 16.38 | 13.78, 21.25 | 13.3, 29.62 | 14.07, 18.31 | 11.96, 20.14 | 13.31, 19.81 |
| 2015 | 13.84, 16.22 | 13.66, 20.62 | 13.21, 29.17 | 13.94, 18.22 | 11.97, 19.94 | 13.15, 20 |
| 2014 | 13.88, 16.26 | 13.67, 20.22 | 13.26, 29.1 | 13.97, 18.34 | 12.07, 19.66 | 13.14, 20.24 |
| 2013 | 13.86, 16.24 | 13.64, 19.91 | 12.75, 28.23 | 13.92, 18.33 | 12.09, 19.54 | 13.13, 20.15 |
| 2012 | 13.93, 16.37 | 13.72, 19.56 | 12.87, 28.22 | 13.99, 18.46 | 12.24, 19.59 | 13.2, 20.22 |
| 2011 | 14.18, 16.53 | 13.88, 19.56 | 13.09, 28.4 | 14.19, 18.76 | 12.5, 19.91 | 13.38, 20.7 |
| 2010 | 14.45, 16.92 | 14.02, 19.2 | 13.38, 28.11 | 14.47, 19.02 | 12.84, 20.26 | 13.64, 20.77 |
| 2009 | 14.54, 17.07 | 14.07, 19.14 | 13.46, 27.38 | 14.56, 19 | 13.04, 20.28 | 13.7, 20.81 |
| **II. Percentage of adverse birth outcomes by demographic characteristics** | | | | | | |
| **Year** | **Education  (Low, Moderate, High)** | | **Marital status (Married, Unmarried)** | **Race (1, 2, 3, 4)*** | | |
| 2023 | 18.09, 15.77, 12.43 | | 13.52, 19.09 | 13.99, 22.49, 17.71, 15.61 | | |
| 2022 | 18.01, 15.76, 12.43 | | 13.55, 19.05 | 13.97, 22.33, 17.81, 15.45 | | |
| 2021 | 18.04, 15.7, 12.31 | | 13.48, 19.05 | 13.9, 22.37, 17.52, 15.42 | | |
| 2020 | 17.61, 15.22, 11.82 | | 13.03, 18.7 | 13.47, 22.1, 17.41, 14.67 | | |
| 2019 | 17.61, 15.31, 12.08 | | 13.24, 18.8 | 13.63, 22.18, 17.02, 14.86 | | |
| 2018 | 17.07, 14.77, 11.64 | | 12.94, 18.46 | 13.16, 21.72, 16.97, 14.18 | | |
| 2017 | 16.97, 14.65, 11.65 | | 12.93, 18.4 | 13.12, 21.47, 17.44, 14 | | |
| 2016 | 16.58, 14.3, 11.6 | | 12.43, 17.26 | 12.95, 21.05, 16.53, 13.8 | | |
| 2015 | 16.27, 14.18, 11.59 | | 12.41, 16.93 | 12.87, 20.95, 16.01, 13.83 | | |
| 2014 | 16.27, 14.13, 11.59 | | 12.44, 16.93 | 12.93, 20.97, 16.37, 13.53 | | |
| 2013 | 16.07, 14.09, 11.72 | | 12.47, 16.8 | 12.93, 20.54, 15.66, 13.33 | | |
| 2012 | 16.11, 14.1, 11.81 | | 12.57, 16.84 | 13.05, 20.63, 16.09, 13.35 | | |
| 2011 | 16.33, 14.23, 11.96 | | 12.79, 17.04 | 13.25, 21.03, 16.16, 13.56 | | |
| 2010 | 16.42, 14.48, 12.33 | | 13.16, 17.19 | 13.57, 21.4, 16.39, 13.94 | | |
| 2009 | 16.31, 14.51, 12.52 | | 13.32, 17.16 | 13.74, 21.5, 16.02, 13.99 | | |
|  | *1=White, 2=Black, 3=AIAN, and 4=Asian or Pacific Islander | | | | | |
